# Sexually transmitted infections among patients with herpes simplex virus at King Abdulaziz University Hospital

**DOI:** 10.1186/1756-0500-6-301

**Published:** 2013-07-31

**Authors:** Wafa M K Fageeh

**Affiliations:** 1 Department of Obstetrics and Gynecology, King Abdulaziz University Hospital, Jeddah, Saudi Arabia

**Keywords:** Herpes simplex virus, HIV, Pregnancy, Fetal outcome, Sexually transmitted infections

## Abstract

**Background:**

Herpes simplex virus (HSV) infection is one of the commonest viral sexually transmitted infections (STIs). The aim of this study was to evaluate the prevalence of STIs among HSV positive patients at a tertiary hospital in Jeddah. Secondary objective of the study included the description of the demographic and clinical profile of patients with HSV and HIV co-infection.

**Methods:**

A retrospective chart review of the medical records was performed for HSV positive women who presented to the emergency room and outpatient department of King Abdulaziz University Hospital, Jeddah, Saudi Arabia between January 1, 2003 and August 30, 2011. Data were collected from the medical records of all the patients and analyzed using the Statistical Package for the Social Sciences.

**Results:**

Three hundred forty-three HSV positive patients were included in this study. Co-infection with HIV was documented in 45 patients (13.1%). Other STIs included chlamydia (n = 43, 12.5%), gonorrhea (n = 44, 12.8%), hepatitis B infection (n = 8, 2.3%), and cytomegalovirus infection (n = 37, 10.8%). Nineteen patients (5.5%) had a total of 47 term pregnancies and five abortions post HSV diagnosis. Genital ulcer disease was diagnosed in 11 (57.9%) of the cases during labor. One newborn developed neonatal herpes infection and subsequently showed delayed psychomotor development during follow up. Genital herpes was diagnosed in one patient’s partner; however, there was no documentation of screening for STIs in the partners of the other patients.

**Conclusions:**

Sexually transmitted infections are relatively common among HSV positive patients at King Abdulaziz University Hospital. Amongst these, HIV is the most common, with a prevalence of 13.1%. Further studies are warranted to evaluate STIs in Saudi Arabia. Health policy makers should adopt a protocol to screen for STIs in the partners of persons who are positive for any STI as early detection and appropriate treatment can improve the outcome.

## Background

Sexually transmitted infections (STIs) are among the most common under diagnosed health problems worldwide, and are rapidly becoming a progressive threat to society. In 2008, there were an estimated 110 million prevalent STIs among women and men in the United States [1]. Amongst these, herpes simplex virus (HSV) infection was reported to be one of the commonest viral STIs. Its incidence continues to rise, especially among females in the reproductive age group [2]. In the Middle East and North African countries, it was estimated that about 9.6 million women aged 15–49 years had HSV infection in 2003 [3]. However, data on other STIs, such as chlamydia, gonorrhea, and syphilis are limited in these countries.

In Saudi Arabia, data on HIV was published for the first time in 2004 [4], and only few reports describe the pattern of STIs in the Kingdom [5,6]. At present, no local studies have been conducted to evaluate the prevalence of herpes simplex virus type 2 (HSV-2); a cause of most genital herpes; and are almost always sexually transmitted. Neither have studies been conducted to estimate the prevalence of herpes simplex virus type 1 (HSV-1), which has emerged as the principal causative agent of genital herpes in some developed countries as well as in the United States [7].

Many studies have confirmed an epidemiologic association between HSV-2 seroprevalence and HIV infection [8-10]. HSV-2 positive patients were found to have an increased risk of acquiring HIV infection, which has been explained by the disruption of the genital mucosa in patients with genital ulcer disease or by decreased immunity in HSV-2 positive persons [11]. Furthermore, it was found that HIV positive patients who have genital ulcers are highly infectious due to HIV shedding from the ulcer site. These patients also tend to have high HIV viral loads [12,13]. Growing data is now describing the benefits of herpes suppression in individuals co-infected with HIV and HSV-2, as herpes suppression lowers HIV viral load even though it would not control HIV sexual transmission [12,14].

Since herpes infection could be asymptomatic but potentially harmful during pregnancy, it is crucial for health care providers to take a good sexual history, especially whenever symptoms or signs raise red flags. However, the stigma associated with STIs, particularly in conservative countries, causes health care providers to be reluctant in taking an explicit sexual history. Hence, a diagnosis of HSV is not reached quite as often as it should. On the one hand, measures such as a more detailed sexual history increase in early diagnosis of HSV infections, screening, as well as proper disease counseling and education will result in decreased disease-related hazards. Routine screening alternatively could be unethical and not cost effective [9].

The purpose of this study is to evaluate the prevalence of STIs among HSV positive patients at a tertiary hospital in Jeddah, Saudi Arabia. Secondary objective was to describe the demographic and clinical profile of patients with HSV and HIV co-infection.

## Methods

A retrospective chart review of the medical records was performed for HSV positive women who presented to the emergency room and outpatient department of King Abdulaziz University Hospital, Jeddah, Saudi Arabia between January 1, 2003 and August 30, 2011.

A total of 406 patients from the initial pool of 546 patients were positive for IgG and / or IgM anti-HSV and/or had a positive viral culture for HSV. Only women, who accounted for 68.7% of all HSV positive cases, were included in the study. The prevalence of positive to negative test ratio in the study population was 3.8 : 1.

Data, including age, presenting complaints, results of laboratory investigations, serious complications, and neonatal outcome in pregnant women were collected from the medical records of all the patients included in the study.

All cases of HSV infection were diagnosed mainly based on clinical suspicion and confirmed by laboratory testing. In all cases, type-specific antibodies to HSV-1 and HSV-2 were determined by an indirect enzyme-linked immunosorbent assay (ELISA). In cases with active lesions, direct detection methods for HSV were performed by viral culture of vesicle fluid and ulcers. HIV cases were also diagnosed based on clinical suspicion. Enzyme-linked immunosorbent assays (R&D Systems Inc., Minneapolis, MN) were used for the detection of HIV-1 and HIV-2. For cases which were positive on ELISA, viral load was determined by highly sensitive quantitative PCR assays. The diagnosis of other STIs, such as syphilis, human papilloma virus infection, chlamydia, gonorrhea, and hepatitis B infection were similarly done based on clinical suspicion and confirmed by laboratory testing. Syphilis was confirmed by rapid plasma regain, human papilloma virus by PCR, chlamydia and gonorrhea by culture, and hepatitis B by ELISA.

### Statistical analysis

Data was analyzed using the Statistical Package for the Social Sciences (SPSS Inc., Chicago, IL, USA). Descriptive statistics of demographic data were performed. Results were expressed as frequency (percent).

Permission to conduct the study was granted by the Ethics Committee of King Abdulaziz University.

## Results

Three hundred forty-three HSV positive patients were included in this study. The mean ± SD age of the patients was 33.5 ± 14.5 years (range, 5 days to 73 years). Of the 343 patients, 76 were Saudis (22.2%); 187 patients (54.5%) were married. Forty-five (13.1%) of the 343 patients had HIV and HSV co-infection. Their mean age was 33.1 ± 18.2 years. Twenty-nine patients (64.4%) were Saudis. Thirty-three (73.3%) were married, while 12 (26.6%) were single. The mean viral load of the patients was 927009 ± 1.8. The mean CD4 count was 543.1 ± 249.1 and CD8 count 1037 ± 110.0. The frequency of other STIs are as shown in Table 1.

**Table 1:** Frequency of other sexually transmitted infections in herpes simplex positive patients

**Infection**	**Frequency**	**Percent**
Hepatitis B	8	2.3
HIV	45	13.1
HPV	16	4.7
CMV IgG	39	11.3
Chlamydia	44	12.8
Gonorrhea	16	4.7

*Abbreviations*: *CMV* cytomegalovirus, *HIV* human immunodeficiency virus, *HPV* human papillomavirus, *HSV* herpes simplex virus.

One hundred sixty-eight patients (49.0%) presented with orofacial lesions, 55 (16.0%) had genital herpes, 52 (15.2%) had encephalitis, nine (2.6%) had cancer, three (0.9%) had multiple sclerosis, and two (0.6%) had systemic lupus erythematosus. Sixty-five patients had a miscellaneous presentation.

Patients with HIV and HSV co-infection (n = 45) presented with the following symptoms: fever (n = 17, 37.8%), myalgia (n = 6, 13.3%), arthralgia (n = 3, 6.7%), loss of appetite (n = 7, 15.6%), nausea (n = 4, 8.9%), vomiting (n = 14, 31.1%), diarrhea (n = 6, 13.3%), weight loss (n = 6, 13.3%), and abdominal pain (n = 11, 24.4%).

Herpes simplex infection was diagnosed in 19 (5.5%) patients during pregnancy. These patients (n = 19; 5.5%) had a total of 47 term pregnancies (mean, 2.4) and five abortions post HSV diagnosis. Genital ulcer disease was diagnosed during labor in 11 (23.4%) of the 47 cases. Of these, seven had vulval warts. Three (15.8%) patients presented with genital ulcers during their first pregnancy. None of the patients received antiviral treatment during pregnancy or delivery.

Vaginal delivery was allowed in four of the eleven cases with active lesions. The average duration of labor in these patients was 5 hours and 5 minutes (range, 6 hours 30 minutes - 12 hours 10 minutes). Cesarean section was performed in the remaining seven cases. Indications for cesarean section included breech presentation (n = 2), twin pregnancy (n = 1), and active genital ulcers (n = 4).

Overall, there were two cases of intrauterine fetal death. Two neonates were positive for HSV IgM antibodies; however, after regular follow-up for two years, these neonates had normal development. Two neonates developed herpetic skin eruption, but they did not show any long-term complications. One newborn developed neonatal herpes infection and subsequently showed delayed psychomotor development during follow up. Genital herpes was diagnosed in one patient’s partner; however, there was no documentation of screening for STIs in the partners of the other patients.

Among the patients with HIV and HSV co-infection, 13 (57.1%) died within one year of diagnosis. Only one patient died during pregnancy.

## Discussion

In a conservative country such as Saudi Arabia, it is considered a taboo to discuss STIs; hence, there are limited data on the prevalence of HSV and other STIs. Local information about the prevalence of HSV and common STIs are available from annually reported cases to the Ministry of Health, and it is believed that the prevalence of HSV infection is considerably underestimated [6]. This is mainly because it is not obligatory to report HSV. More so, many patients with HSV infection may not seek medical advice, as awareness about HSV is markedly deficient [15].

In this study, we evaluated the prevalence of STIs, including HIV, in HSV positive patients who were followed up at King Abdulaziz University Hospital. Our findings demonstrated that 13.1% of HSV positive patients had HIV co-infection. The prevalence of other STIs, namely hepatitis (2.3%), human papillomavirus (4.7%), cytomegalovirus (11.3%), chlamydia (12.8%), and gonorrhea (4.7%) were relatively low. This low prevalence is mainly because we do not screen for STIs, and testing is restricted to patients complaining of symptoms suggestive of an STI.

In a recent study, an estimated 22% of pregnant women are reported to have HSV infection, and up to 80% of the women who have genital herpes are unaware of being infected [16]. In the current study, HSV infection was diagnosed in 5.5% of the women during pregnancy, and genital herpes was diagnosed in 57.9% of the cases. Although the incidence of HSV transmission from mother to child is <1% even when the virus is detectable in the maternal genital tract at the time of delivery [17], it is imperative to diagnose the infection early, as delayed diagnosis and initiation of therapy contributes to poor neonatal outcome [18].

Overall, there were two cases of intrauterine fetal death, two neonates developed herpetic skin eruption without long-term complications, one case of neonatal herpes infection with subsequent delayed psychomotor development, three cases of congenital anomaly, and five cases of abortion. Even though intrauterine HSV infection is associated with hydrops fetalis and fetal death, and surviving infants of *in utero* HSV infection have symptoms at birth similar to other congenital infections, other etiologies, namely chlamydia, syphilis, hepatitis, and CMV infection, which are associated with poor pregnancy and neonatal outcomes [19-22], cannot be excluded.

According to the American College of Obstetricians and Gynecologists, women with active recurrent genital herpes should be offered suppressive viral therapy at or beyond 36 weeks of gestation. Cesarean section should be performed in women with active genital lesions or symptoms, such as vulvar pain or burning at delivery, because these symptoms may indicate an impending outbreak [23]. However, none of our patients received antiviral treatment during pregnancy or delivery. More so, four of the patients with active disease were delivered vaginally, and cesarean section was only performed in four cases for active genital lesions. This may be due to lack of awareness about STIs in both patients and health care practitioners, as all cases of GUD were only diagnosed during labor. In a conservative community such as ours, partners of persons with STIs are usually not screened and physicians do not take adequate sexual histories or perform a complete physical examination. It is not uncommon for women to try herbal treatment for infections, especially when these involve the genitalia [24,25].

Data from the National AIDS Program of the Ministry of Health in Saudi Arabia shows that the prevalence of HIV is higher among non-Saudis [26]. However, there are limited data on the prevalence of HIV in hospital-based studies, and two studies conducted in tertiary hospitals in Riyadh reported a prevalence of zero percent among Saudis and expatriates [27,28]. In the current study, HIV infection was less frequent among non-Saudis. Given that it is requirement of the Saudi authorities that all expatriates entering the country undergo rigorous medical screening for HIV and the hepatitis viruses, it is plausible that these patients acquired HIV infection while in Saudi Arabia. According to one report, the main mode of HIV transmission in Saudi Arabia was heterosexual contact, and most of the women in the study reportedly acquired the infection either through blood transfusion or marital sex with their infected husbands [5]. However, in other cases with unidentifiable risk factors, it was suggested that the patients acquired the infection through illegal or non-marital sex [5].

Non-specific symptoms, such as fever, myalgia, vomiting, diarrhea, and abdominal pain were common in our patients with HSV and HIV co-infection. It has been reported that primary infection may be complicated by systemic symptoms, such as fever, headache, and myalgia (38% in men, 68% in women) [29], and pre-existing HSV-1 antibodies can alleviate clinical manifestations of subsequently acquired HSV-2 [6]. Systemic clinical findings may be the only presenting symptom of infection in some patients, and in over half of the cases, primary infection goes unnoticed [29].

Some limitations of our study warrant consideration. First, it was limited by its retrospective nature. Second, because of the difficulty in diagnosing chlamydia and gonorrhea infection in women [30,31], some cases may have been missed as no policy exists in our institution for testing and diagnosing these infections. Third, the long study period and the wide range of age of the patients should be taken into consideration when interpreting the results. Nonetheless, this study is the first to report the prevalence of STIs among HSV positive patients at a university hospital in Jeddah, Saudi Arabia.

## Conclusions

Sexually transmitted infections are relatively common among HSV positive patients at King Abdulaziz University Hospital. Amongst these, HIV is the most common, with a prevalence of 13.1%. Most patients with HSV and HIV infection present with non-specific symptoms and therefore diagnosis is mainly based on clinical suspicion contributing to its underreporting.

Further studies are necessary to evaluate STIs in Saudi Arabia. In order to provide appropriate treatment and consequently minimize the spread of infection, suspected cases should be screened for common STIs. Health policy makers should also adopt a protocol to screen for STIs in the partners of persons who are positive for any STI, as early detection and appropriate treatment can improve the outcome.

## Competing interests

The author declares that he has no competing interests.

## Authors’ contributions

As the sole author of this paper, WMKF conceived and designed the study, analyzed and interpreted the data, drafted the manuscript and revised it for important intellectual content, and gave the final approval for the version to be published.
